# The immune-regulating effect of Xiao’er Qixingcha in constipated mice induced by high-heat and high-protein diet

**DOI:** 10.1186/s12906-017-1700-9

**Published:** 2017-03-31

**Authors:** Chang Qu, Guang-Hua Yang, Rong-Bo Zheng, Xiu-Ting Yu, Shao-Zhong Peng, Jian-Hui Xie, Jian-Nan Chen, Xiu-Fen Wang, Zi-Ren Su, Xiao-Jun Zhang

**Affiliations:** 1 School of Chinese Materia Medica, Guangzhou University of Chinese Medicine, Guangzhou, 510006 People’s Republic of China; 2 The First Affiliated Hospital of Chinese Medicine, Guangzhou University of Chinese Medicine, Guangzhou, 510450 People’s Republic of China; 3 Guangzhou Wanglaoji Pharmaceutical Company Limited, Guangzhou, 510005 People’s Republic of China; 4 Guangdong Provincial Key Laboratory of Clinical Research on Traditional Chinese Medicine Syndrome, The Second Affiliated Hospital, Guangzhou University of Chinese Medicine, Guangzhou, 510120 People’s Republic of China; 5 Guangdong Provincial Key Laboratory of New Chinese Medicinal Development and Research, Guangzhou, 510006 People’s Republic of China; 6 Dongguan Mathematical Engineering Academy of Chinese Medicine, Dongguan, 523000 People’s Republic of China

**Keywords:** Xiao’er Qixingcha, Constipation, High-heat and high-protein diet, Laxative, Mucosal barrier, Immunity function

## Abstract

**Background:**

Xiao’er Qixingcha (EXQ) has been extensively applied to relieve dyspepsia and constipation in children for hundreds of years in China. However, the therapeutic mechanism underlying its efficacy remained to be defined. The present study aimed to clarify the possible laxative and immune-regulating effects of EXQ on two models of experimental constipation in mice, which mimicked the pediatric constipation caused by high-heat and high-protein diet (HHPD).

**Methods:**

The two models of constipated mice were induced by HHPD or HHPD + atropine respectively. To investigate the laxative and immune-regulating activities of EXQ, animals were treated with three doses of EXQ (0.75, 1.5 and 3 g/kg) for 7 consecutive days. The fecal output parameters (number and weight), weight of intestinal content and, the thymus and spleen indexes were measured. The levels of sIgA, IL-10, TNF-α and LPS in colon and serum were determined by ELISA. Furthermore, the pathological changes of colon tissue were examined after routine H&E staining.

**Results:**

Both HHPD and HHPD + atropine treatments obviously inhibited the fecal output and reduced the colonic sIgA, prominently increased the levels of IL-10 and TNF-α in colonic tissue and elevated the contents of LPS in serum and colonic tissues. In contrast, oral administration of EXQ significantly improved the feces characters and dose-dependently decreased the intestinal changes in both models. In HHPD model test, EXQ efficaciously boosted the sIgA level in a dose-dependent manner, significantly elicited decreases in TNF-α and IL-10 levels, and evidently decreased the spleen and thymus indexes. In HHPD + atropine model test, EXQ treatment reversed the pathological changes by not only dramatically decreasing the spleen index and the levels of LPS and IL-10, but also markedly elevating the thymus index. Furthermore, microscopic observation revealed that EXQ treatment maintained the integrity of colonic mucosa, and protected the colonic tissues from inflammation in the both models.

**Conclusions:**

EXQ exhibited prominent laxative activity and effectively protected the colonic mucosal barrier in two models of constipated mice, of which the mechanism might be closely associated with its propulsive and immune-regulating properties. The current results not only validated the rationale for the clinical application of EXQ in pediatric constipation related symptoms, but also threw new light on the immune-inflammatory responses accompanied with chronic constipation pathology.

## Background

Constipation is a common gastrointestinal disorder, characterized by symptoms such as straining, hard stool, and infrequent defecation [1]. Nowadays, it’s also a considerable concern for children, with up to 30% being affected [2]. Constipation may lead to a painful defecation, and children will conflict with defecation to avoid a repeated painful experience, which may aggravate constipation [3]. It not only causes physical and mental suffering for children such as abdominal distension, vomiting, gut obstruction and perforation, but also has adverse effects on their quality of life by increasing the risk of anxiety, irritability and decreasing appetite [4, 5].

Constipation may result of dietary, metabolic and painful anorectal condition; however, unhealthy dietary habits are most common cause in children [6, 7]. As the life quality improved, parents tend to seek refined and high-nutrition diets for their children. Many valuable nutrients, such as fibers, are refined out of the foods during processing. On the other hand, children’s digestive system is immature, thus the digestive ability and intestine propulsion are weak in comparison with adults. Therefore, long-term high-heat and high-protein diet (HHPD) could further increase the gastrointestinal burden and cause disorder, even intractable constipation.

Nowadays, many types of synthetic drugs have been used to relieve pediatric constipation, but their adverse effects have also raised public concern [8, 9]. As a complementary therapy, traditional herbal medicines have been used with apparent safety and efficacy [10]. For example, herbal formulations, like Ma Zi Ren Wan, are gaining considerable attention for the treatment of constipation in recent years [11, 12]. Xiao’er Qixingcha (EXQ), a well-known pediatric multi-herbal patent medicine in Hong Kong and mainland China, has been extensively applied to relieve pediatric disorders due to dyspepsia, poor appetite and constipation, and is especially suitable for disorders caused by HHPD [13, 14]. EXQ contains seven herbs with a specific ratio, namely *Semen coicis*, *Fructus oryzae germinatus*, *Fructus crataegi*, *Lophatherum gracile*, *Uncaria rhynchophylla*, *Cicada slough* and *Glycyrrhiza uralensis* (Table 1) [13], in which *Glycyrrhiza uralensis* and *Fructus crataegi* have been reported to have laxative effects [15, 16]. According to the statistical data of Guangzhou Wanglaoji Pharmaceutical Co. Ltd., the largest manufacturer of EXQ, the sale values of EXQ reached RMB 198 million in 2009, and exceeded 300 million in 2013. However, despite the extensive application of EXQ, its effects and underlying mechanism remain obscure.

**Table 1:** Components of Xiao’er Qixingcha (EXQ)

Plant name	Plant part	Weight (g)	Voucher number
*Semen Coicis* (*Coix lacryma-jobi* L. var. *mayuen* (Roman.) Stapf)	Fruit	893	EXQ160505-A01
*Fructus oryzae germinatus* (*Oryzasativa* L.)	Fruit	893	EXQ160505-B02
*Crataegi Fructus* (*Crataegus pinnatifida* Bge.)	Fruit	446	EXQ160505-C03
*Lophatherum gracile* (*Lophatherum gracile* Brongn.)	Leaf	670	EXQ160505-D04
*Uncaria tomentosa* (*Uncaria rhynchophylla* (Miq.) Miq. ex Havil.)	Hook-bearing stems and branch	335	EXQ160505-E05
*Periostracum Cicadae* (*Cryptotympama pustulata* Fabricius)	cast-off shell of insects	112	EXQ160505-F06
*Glycyrrhiza uralensis* (*Glycyrrhiza uralensis* Fisch.)	Root and rhizome	112	EXQ160505-G07

Our previous study revealed that EXQ exhibited prominent prokinetic and laxative activities on diphenoxylate-induced and water-fasted-induced mice constipation [14]. Studies have suggested that unhealthy diets, especially HHPD and low-fiber diet are crucial predispositions in pediatric constipation [7, 17]. And, one striking characteristic of chronic constipation is low-grade inflammation in mucosa [18], which is frequently observed after low-fiber diets [19]. On the other hand, colonic inflammation caused dysmotility and significant impairment of propulsion in animals [20, 21]. Thus, it is possible that the deficit in propulsive activity and associated fecal retention resulting from unhealthy diets would cause intestinal inflammation, and once the tissue damage occurred, it might exacerbate the motility disturbance.

Therefore, in this experimental study, we aimed to further investigate whether EXQ could relieve HHPD-induced constipation by promoting propulsion and defecation, and whether EXQ would remit the concurrent intestinal inflammation. To achieve this, we examined the effects of EXQ on two models of constipated mice, namely ‘HHPD model’ and ‘HHPD + atropine model’, which mimicked the symptoms caused by HHPD alone and HHPD in combination with deficient gastrointestinal motility respectively, as observed in constipated children.

## Methods

### Preparation of EXQ

EXQ extract, composed of 7 kinds of medicinal herbs (Table 1), was produced by Guangzhou Wanglaoji Pharmaceutical Co. Ltd. (Batch No. 121104). These herbs were purchased from Qingping Chinese herbal medicine market of Guangzhou (Guangdong, China), and authenticated by Professor Chen Jian-nan, School of Chinese Materia Medica, Guangzhou University of Chinese Medicine (GZUCM). The authenticated voucher specimens were deposited at TCM research laboratory of School of Chinese Materia Medica, GZUCM, with voucher specimen numbers shown in Table 1. Assurance of quality control for all the materials was validated according to the Chinese Pharmacopeia (2015 edition).

The extraction process was performed as follows [13]: briefly, *Semen Coicis* and *Fructus oryzae germinatus* were extracted with boiling water for 2 h twice. After filtration, the aqueous extract was concentrated to a relative density (RD) of 1.08-1.12 (55 °C) by rotary evaporator under vacuum. Ethanol was added into the concentrated extract until the percentage of ethanol was amounted to 45%, and then stored at 4 °C. The obtained extract was centrifuged for 15 min at 3000 rpm and the supernatant was concentrated to obtain thick paste. The rest herbs were extracted twice with distilled water at 100 °C for 2 h. The resulting extract was filtered and concentrated to an appropriate amount, and then blended with the aforementioned thick paste. EXQ (3.75, 7.5 and 15 g) was dissolved in distilled water to the volume of 100 mL to prepare the desired concentrations.

HPLC assays as described previously had been employed for the quality control of the typical chemicals of the herbs, liquiritin for *Lophatherum gracile*, *Uncaria tomentosa* and *Glycyrrhiza uralensis*, chlorogenic acid for *Crataegi Fructus* and *Uncaria tomentosa*, and apigenin for *Glycyrrhiza uralensis* and *Uncaria tomentosa*, respectively [22].

### Chemical and reagents

Mouse secretory immunoglobulin A (sIgA) and lipopolysaccharide (LPS) kits were purchased from Beijing Chenglin Biotechnology Company. Mouse TNF-α and IL-10 kits were purchased from eBioscience (CA, USA). PBS and Tween-20 were obtained from Thermo-Fisher Sci. Co. Ltd. (MA, USA). Milk powder, soybean meal, flour and dried fish floss were obtained from Jingdong Online Mall. All other chemicals used in this study were of analytical grade.

### Animals

Male KM mice (10.0 ± 2.0 g), aged about 3 weeks, were purchased from the Experimental Animal Center of Guangzhou University of Chinese Medicine (Approval number SCXK (Guangzhou)-2013-0020). The animals were housed under standard environmental conditions (22 ± 1 °C, humidity 50-70%, 12 h light/dark cycle) with free access to standard diet and water. All animal procedures were performed according to the Guide for the Care and Use of Laboratory Animal of the National Institute of Health as well as Guide of the Animal Welfare Act and approved by the Animal Ethics Committee of Guangzhou University of Chinese Medicine (approval No. SCXK2015043).

### Preparation of feed

Standard mice pellet feed was provided by Experimental Animal Center of Guangzhou University of Chinese Medicine, China. High-heat and high-protein diet was prepared as follows [23, 24]: milk powder, dried fish floss, flour and soybean meal were weighed according to the ratio of 1: 1: 1: 2, and then mixed homogeneously with water and dehydrated at 50 °C to manufacture the HHPD pellet feed. High-heat and high-protein solution was made from the milk powder and water (52: 48) mixture before administration.

### Experimental procedures

The potential beneficial effect of EXQ were tested on two constipated mice models which mimic unhealthy diet and insufficient gastrointestinal mobility in pediatric constipation, namely HHPD model and HHPD + atropine model. Atropine, a classic anti-cholinergic agent, could inhibit intestinal peristalsis, slow down gastrointestinal transit, contract arteriole in intestinal mucosa, and thus reduces secretion and defecation [25, 26]. As reported, atropine was applied to induce constipation in murine model, mimicking clinical constipation [27]. Hence, in this study, atropine was applied to retard gastrointestinal mobility and aggravate constipation in murine model.

#### In-vivo experiment in HHPD model

After 1 week of acclimatization, 40 mice were randomly divided into 5 groups (*n* = 8 per group): normal control group, vehicle group, and EXQ treatment (0.75, 1.5 and 3 g/kg) groups. The model was established according to the method previously described [23, 24, 28], with minor modifications. Briefly, vehicle group and EXQ-treated groups were provided with high-heat and high-protein feed, and simultaneously orally administrated with 52% milk solution (0.2 mL/10 g) twice a day, to induce constipation. Subsequently, EXQ-treated groups were orally administrated with EXQ at doses of 0.75, 1.5 and 3 g/kg once daily, while the mice in normal control group and vehicle group received an equal volume of distilled water for 7 days. On the 8th day, mice were placed in small metabolic cages individually for adaptation. On the 10th day, feces were collected, counted and weighed. Subsequently, the blood samples were collected from the retroorbital venous plexus of mice with a heparinized glass capillary. Then mice were sacrificed and opened longitudinally. The duodenum from pylorus to caecum, about 8 cm, was quickly obtained, weighed and dissected, then washed in ice-cold physiological saline, blotted on filter paper and weighed again. The weight of intestinal content was calculated as the difference between the two weights. The colon segments were also harvested, rinsed with ice-cold physiological saline, blotted on filter paper and reserved. The dose selection in the present work was mainly based on our previous investigation [14], the Chinese pharmacopeia [13] and our pilot trial.

#### In-vivo experiment in HHPD + atropine model

Animal grouping and administration were in accordance with the above HHPD model. The experiment was carried out according to the previous method with the following modifications [23, 24, 28]. During the experimental period, vehicle and EXQ treatment groups were orally administrated with 52% milk solution (0.2 mL/10 g) and intraperitoneally with atropine (0.02 mg/10 g) twice daily. All other experimental procedures were performed in line with the HHPD model.

### Measurement of immune organ index

On the 10th day, mice were sacrificed by cervical dislocation, and the thymus and spleen were gathered quickly for weighing. The calculation was carried out according to the following formulas: Spleen index = spleen weight (g) /body weight of mice (g) × 1000 (mg/g); Thymus index = thymus weight (g) /body weight of mice (g) × 1000 (mg/g).

### Measurement of cytokines

The colonic tissue samples were stored at 2-8 °C, cut into about 1 mm^3^ sections, embedded in a certain quantity phosphate-buffered saline (PBS) solution (pH 7.4), fully homogenized, and then centrifuged for 20 min (12,000 rpm, 4 °C). The serum samples were solidified naturally for 20 min at room temperature, and then centrifuged (3000 rpm, 4 °C) for 20 min. The supernatants in the colonic tissue and serum were used to assess the secreted levels of TNF-α and IL-10 by ELISA kits following the manufacturer’s instructions.

### Measurement of sIgA and LPS

The supernatants in the colonic tissue and serum were prepared as described above for the measurement of sIgA and LPS using the corresponding ELISA kits following the manufacturer’s instructions.

### Histological examination

The harvested colonic tissues were fixed in 4% paraformaldehyde, dehydrated through graded alcohol, embedded in paraffin, sectioned at a thickness of 5 mm, and then stained with hematoxylin and eosin (H&E) according to standard procedures for a general morphologic assessment after deparaffinization. Then, the stained slides were mounted in neutral balsam and covered with coverslips [29]. Histopathologic changes were observed under optical microscopy (BX53 Olympus, Japan).

### Statistical analysis

All values were expressed as means ± standard deviation (SD). Statistical analysis was performed using Statistical Analysis Software (SPSS Inc., Chicago, IL) (SPSS 19.0). The data were analyzed by one-way ANOVA followed by the Dunnett’t test, and the differences were considered statistically significant when *P* < 0.05.

## Results

### EXQ ameliorated fecal output character and intestinal content in two models of constipated mice

**Fig. 1:**
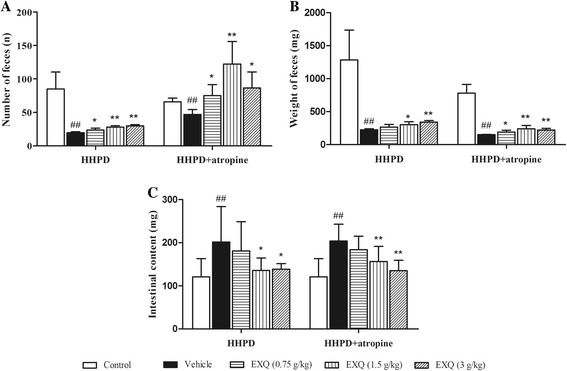
Effect of EXQ on intestinal mobility in two models of experimental constipated mice. Mice were orally given EXQ at the doses of 0.75, 1.5 and 3 g/kg, respectively. **a** number of feces; **b** weight of feces; **c** weight of intestinal content. Data are presented as the mean ± SD (*n* = 8). #*P* < 0.05, ##*P* < 0.01 compared to the normal control group; * *P* < 0.05, ** *P* < 0.01 compared to the vehicle group

To investigate whether EXQ exhibited laxative effects on constipated mice, we firstly examined the effect of EXQ on the fecal output (number and weight) and weight of intestinal content (Fig. 1), which were direct indicators of constipation in animals. In HHPD model test, as compared to the normal control group (number: 85.0 ± 25.5; weight: 1284.9 ± 454.5 mg; intestinal content: 120.6 ± 42.6 mg), the number (19.6 ± 1.3) and weight (225.9 ± 14.7 mg) of feces in the vehicle group were significantly decreased (*P* = 0.001, *P* = 0.002, respectively), and the weight of intestinal content (206.6 ± 81.9 mg) were notably increased (*P* = 0.006). By contrast, EXQ administration counteracted the decreases or retention induced by HHPD in a dose-dependent manner. EXQ at the doses of 1.5 and 3 g/kg significantly increased the fecal number by 43.6 and 51.6%, and weight by 34.5 and 51.6%, respectively. Likewise, EXQ treatments at 1.5 and 3 g/kg significantly decreased the weight of intestinal content (135.5 ± 30.0 and 138.4 ± 13.0 mg; for both groups *P* = 0.033 and *P* = 0.034 vs. vehicle group, respectively).

In HHPD + atropine model test, as compared with the normal control group (number: 65.7 ± 5.9; weight: 780.6 ± 132.6 mg; intestinal content: 120.6 ± 42.6 mg), the number (46.9 ± 7.4) and weight (151.5 ± 1.9 mg) of feces in the vehicle group were significantly decreased (*P* = 0.001, *P* = 0.000, respectively), whereas the weight of intestinal content (203.8 ± 38.8 mg) was increased substantially (*P* = 0.000). By contrast, EXQ attenuated the decrease or retention induced by HHPD + atropine. The three doses of EXQ elevated the fecal number by 60.6, 161.2 and 84.6%, and increased the fecal weight by 26.4, 59.9 and 46.7%, respectively. Moreover, EXQ at the doses of 1.5 and 3 g/kg remarkably decreased the weight of intestinal content (156.4 ± 35.1 mg; 135.0 ± 24.1 mg; for both *P* = 0.010, *P* = 0.000 vs. vehicle group). Hence, the results obtained indicated that EXQ treatment could facilitate the fecal output in both HHPD and HHPD plus atropine models.

### EXQ regulated the indexes of spleen and thymus in two models of constipated mice

**Fig. 2:**
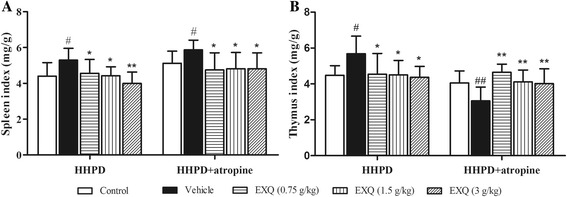
Effects of EXQ on immunity function in two models of experimental constipated mice. **a** spleen index; **b** thymus index. Data are shown as mean ± SD (*n* = 8). #*P* < 0.05, ##*P* < 0.01 compared to the normal control group; * *P* < 0.05, ** *P* < 0.01 compared to the vehicle group

Thymus and spleen are two major immune organs and their indexes are preliminary indicators to assess the body immune status [30]. To investigate whether the experimental constipation was associated with inflammatory response in mice, and whether the laxative effect of EXQ was related to the immune-regulation in constipated mice, the spleen and thymus indexes were measured (Fig. 2).

In HHPD model test, as compared to the normal control group (spleen index: 4.40 ± 0.75 mg/g; thymus index: 4.49 ± 0.53 mg/g), the spleen index (5.30 ± 0.66 mg/g) and the thymus index (5.68 ± 0.98 mg/g) in the vehicle group were significantly increased (*P* = 0.020 and *P* = 0.026, respectively). Conversely, the spleen index (4.56 ± 0.78, 4.43 ± 0.50 and 4.00 ± 0.64 mg/g; for all *P* = 0.045, *P* = 0.029, *P* = 0.001 vs. vehicle group, respectively) and thymus index (4.55 ± 1.15, 4.50 ± 0.81 and 4.38 ± 0.59 mg/g; for all *P* = 0.029, *P* = 0.033, *P* = 0.025, respectively) in three EXQ-treated groups were significantly decreased as compared with the corresponding values in the vehicle group, respectively.

In HHPD + atropine model test, when compared to the normal control group (spleen index: 5.12 ± 0.66 mg/g; thymus index: 4.05 ± 0.66 mg/g), the spleen index (5.87 ± 0.54 mg/g) was significantly increased and the thymus index (3.05 ± 0.77 mg/g) was obviously decreased in the vehicle group (*P* = 0.032, *P* = 0.006, respectively). However, in three EXQ groups (0.75, 1.5 and 3 g/kg), the spleen index was remarkably decreased (4.75 ± 0.96, 4.81 ± 0.91 and 4.82 ± 0.90 mg/g; for all *P* = 0.022, *P* = 0.011, *P* = 0.011, respectively vs. vehicle group) and the thymus index was efficaciously increased (4.65 ± 0.45, 4.11 ± 0.66 and 4.02 ± 0.82 mg/g, for all *P* = 0.000, *P* = 0.004 and *P* = 0.008 vs. vehicle group), respectively.

### EXQ elevated the levels of colonic sIgA in two models of constipated mice

sIgA is the predominant immunoglobulin secreted at the intestinal mucosal surfaces and is a critical mediator of mucosal immune response. Besides, sIgA can protect mucosal surfaces from microbial invaders [31, 32]. To further explore the protective mechanism of EXQ on epithelium mucosa upon HHPD or HHPD plus atropine challenge, the mucosal sIgA concentration was measured.

**Fig. 3:**
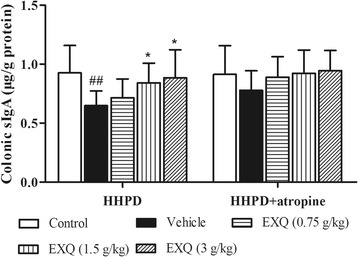
Effects of EXQ on sIgA production in two models of experimental constipated mice. Data are shown as mean ± SD (*n* = 8). #*P* < 0.05, ##*P* < 0.01 compared to the normal control group; * *P* < 0.05, ** *P* < 0.01 compared to the vehicle group

As shown in Fig. 3, HHPD resulted in a significant inhibition on the level of colonic sIgA in the vehicle group (0.65 ± 0.12 μg/g, *P* = 0.006), as compared with the normal control group (0.93 ± 0.23 μg/g). However, EXQ treatment increased the level of sIgA in a dose-dependent manner. Significant elevations were observed at the doses of 1.5 and 3 g/kg (0.84 ± 0.17 and 0.88 ± 0.24 μg/g, for both *P* = 0.049, *P* = 0.018 vs. HHPD group, respectively), presenting ascending amplitudes of 29.8 and 36.3% over the corresponding value of HHPD group, respectively.

The administration of HHPD + atropine also caused a decrease in the level of sIgA (0.91 ± 0.24 μg/g), when compared with the normal control group (0.78 ± 0.17 μg/g). After EXQ treatment, the sIgA levels exhibited a trend of increase, but the difference didn’t achieve statistical significance.

### EXQ regulated the levels of IL-10 in two models of constipated mice and TNF-α in HHPD mice

**Fig. 4:**
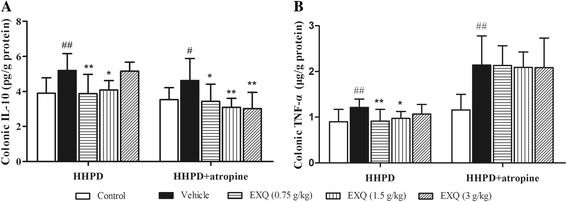
Effects of EXQ on IL-10 and TNF-α levels in two models of experimental constipated mice. The levels of IL-10 (**a**) and TNF-α (**b**) were determined by ELISA. Data are presented as mean ± SD (*n* = 8). #*P* < 0.05, ##*P* < 0.01 compared to the normal control group; * *P* < 0.05, ** *P* < 0.01 compared to the vehicle group

Chronic constipation is closely related to the mucosa inflammation. To investigate whether the protective effect of EXQ was associated with the anti-inflammatory effect in constipated mice, the levels of colonic TNF-α and IL-10 were measured (Fig. 4).

In HHPD model test, the levels of IL-10 (5.20 ± 0.95 pg/g) and TNF-α (1.22 ± 0.18 pg/g) in the vehicle group were significantly elevated (*P* = 0.004, *P* = 0.007, respectively) as compared to the normal control group (IL-10: 3.99 ± 0.89 pg/g, TNF-α: 0.90 ± 0.27 pg/g). However, treatment of EXQ substantially attenuated the abnormal productions of IL-10 and TNF-α when compared with the vehicle group, especially at the doses of 0.75 and 1.5 g/kg.

In HHPD + atropine model test, when compared to the normal control group (IL-10: 3.54 ± 0.68 pg/g, TNF-α: 1.16 ± 0.34 pg/g), there were significant increases in colonic levels of IL-10 (4.63 ± 1.25 pg/g, *P* = 0.021) and TNF-α (2.14 ± 0.64 pg/g, *P* = 0.000). In contrast, EXQ treatment distinctly decreased the IL-10 level (3.43 ± 0.98, 3.09 ± 0.51 and 3.02 ± 0.92 pg/g; both *P =* 0.012, *P =* 0.002, *P =* 0.001, respectively). However, its effect on the production of TNF-α failed to research statistical significance.

### EXQ decreased the levels of colonic and serum LPS in constipated mice induced by HHPD + atropine

Defected intestinal mucosal barrier was closely related to the diseases of gastrointestinal tract, including constipation. And the mucosal barrier function of the intestine was associated with the bacterial component LPS from the intestine, which can trigger inflammatory reactions [33–36]. Hence, LPS is deemed as one of the important factors that contribute to the mucosal barrier function. To investigate whether EXQ exhibited the protective effect on intestinal mucosal barrier in constipated mice, the levels of colonic and serum LPS were investigated.

**Fig. 5:**
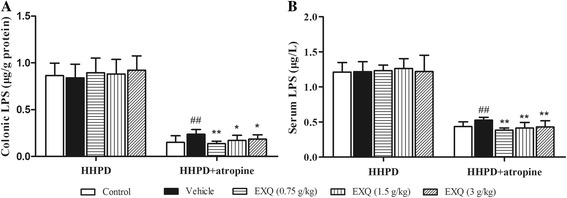
Effect of EXQ on colonic and serum LPS levels in two models of experimental constipated mice. The levels of colonic LPS (**a**) and serum LPS (**b**) were determined. Data are presented as mean ± SD (*n* = 8). #*P* < 0.05, ##*P* < 0.01 compared to the normal control group; * *P* < 0.05, ** *P* < 0.01 compared to the vehicle group

As shown in Fig. 5, in HHPD model test, no significant changes were observed among the five groups. However, in HHPD + atropine model test, the LPS contents in the colon (0.24 ± 0.05 μg/g) and serum (0.53 ± 0.04 μg/L) of the vehicle group were significantly increased as compared to the normal control group (colon: 0.15 ± 0.07 μg/g, serum: 0.44 ± 0.07 μg/L, for both *P* = 0.002, *P* = 0.009). On the contrary, the three doses of EXQ effectively decreased the levels of colonic LPS (0.14 ± 0.03, 0.17 ± 0.05 and 0.19 ± 0.05 μg/g, for all *P* = 0.000, *P* = 0.015, *P* = 0.050, respectively) and serum LPS (0.39 ± 0.03, 0.41 ± 0.08 and 0.43 ± 0.09 μg/L, for all *P* = 0.000, *P* = 0.002, *P* = 0.006 vs. vehicle group, respectively) in comparison to the vehicle group.

### EXQ ameliorated the histological changes in two models of constipated mice

To further analyze the effect of EXQ treatment on the histological structure of the transverse colon, alterations in several histological parameters were measured in H&E staining.

**Fig. 6:**
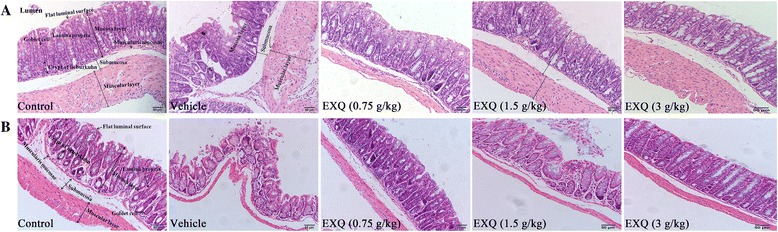
Histological examinations for the possible protective effect of EXQ in two models of experimental constipated mice (Original magnification ×200). **a** HHPD model; **b** HHPD + atropine model

As depicted in Fig. 6a, the colon sections of normal control group exhibited normal morphologic characteristics of epithelial cells, well-demarcated crypt and goblet cells. In contrast, HHPD treatment resulted in a dramatic damage in the colonic mucosal structural integrity, characterized by randomly arranged or lost monolayer columnar epithelia and marked inflammatory cell infiltration into mucosa. Furthermore, and the thicknesses of mucosa, submucosa and muscle were significantly shorter in the vehicle group than the control group (Table 2). However, these pathological changes were notably ameliorated by EXQ, and the thicknesses of mucosa, submucosa and muscle rapidly increased by 26.55, 42.82 and 43.47% following EXQ treatment at the dose of 3 g/kg as compared with the vehicle group (Fig. 6a). In addition, the numbers of goblet cells and crypt of lieberkuhn were 32.21 and 47.92% lower in the vehicle group than the normal control group, respectively (Table 2). However, these parameters were markedly increased in the EXQ middle dose (16.61 and 31.14%) and EXQ high dose (22.96 and 43.97%) groups (Table 2). Additionally, high dose of EXQ completely recovered the numbers of goblet cells and crypt of lieberkuhn to those of the normal control group. Also, the mucosa was largely integrated and only slight infiltration of inflammatory cells was observed in the EXQ groups. Similar beneficial effect was also observed in the EXQ groups in HHPD + atropine model test (Fig. 6b).

**Table 2:** Histopathological alteration of the constipated mice after EXQ treatment

Categories	Model	Group				
		Control	Vehicle	EXQ (0.75 g/kg)	EXQ (1.5 g/kg)	EXQ (3 g/kg)
Mucosa thickness (μm)	HHPD	235.38 ± 30.19	190.78 ± 29.65^#^	262.05 ± 37.12^*^	264.11 ± 53.57^*^	259.74 ± 37.02^*^
	HHPD + atropine	174.19 ± 34.58	167.81 ± 27.52	211.72 ± 9.68^*^	219.92 ± 49.80^*^	217.93 ± 34.63^*^
Muscle thickness (μm)	HHPD	146.44 ± 24.67	80.20 ± 17.38^##^	106.03 ± 26.40	149.91 ± 18.93^**^	140.25 ± 37.16^**^
	HHPD + atropine	48.90 ± 4.85	24.79 ± 10.73^#^	57.33 ± 19.95^**^	48.39 ± 6.68^*^	51.88 ± 21.31^*^
Submucosa thickness (μm)	HHPD	46.37 ± 9.46	27.15 ± 5.46^#^	41.00 ± 14.75	54.65 ± 16.31^**^	48.03 ± 12.07^*^
	HHPD + atropine	44.84 ± 15.86	30.04 ± 5.64	31.60 ± 5.01	41.52 ± 15.22	41.78 ± 14.67
Number of goblet cell (ea)	HHPD	376.17 ± 29.03	255.00 ± 31.03^##^	289.17 ± 27.09^*^	305.80 ± 28.47^**^	331.00 ± 23.82^**^
	HHPD + atropine	210.86 ± 44.95	109.80 ± 33.49^##^	190.00 ± 52.35^**^	188.00 ± 36.99^**^	220.83 ± 34.14^**^
Number of crypt of lieberkuhn (ea)	HHPD	31.20 ± 4.55	16.25 ± 2.22^##^	24.00 ± 6.04^*^	23.60 ± 4.45^*^	29.00 ± 5.79^**^
	HHPD + atropine	27.00 ± 5.26	17.00 ± 3.32^##^	27.00 ± 8.49^*^	25.20 ± 6.72^*^	27.17 ± 5.38^**^

^#^ *P* < 0.05, ^##^ *P* < 0.01 indicates significance when compared with the normal control group

^*^ *P* < 0.05, ^**^ *P* < 0.01 indicates significance when compared with the vehicle treated constipation group

The aforementioned results indicated that treatment of EXQ effectively protected against HHPD or HHPD + atropine induced mucosal injuries and attenuated the deteriorated histological changes in constipated mice.

## Discussion

EXQ is a famous household herbal formula to relieve pediatric constipation for children and infants in China. Our previous investigation has clearly demonstrated that EXQ effectively stimulated intestinal peristalsis and improved the fecal output in diphenoxylate-induced and water-fasted-induced mice constipation [14]. In the present work, we chose 3-week old mice to establish the pediatric constipation model, and investigate the potential laxative effect and mechanism of EXQ. According to the Chinese pharmacopeia [13] and the body surface area based dose translation equation [37], the clinical equivalent dose for mice is 1.5 g/kg. Therefore, this dose was selected as the middle dose. Hence, the doses of 0.75, 1.5, 3 g/kg was employed in the current investigation.

Previous studies have suggested that unhealthy diets involving HHPD and low-fiber diet predisposed children to constipation [7, 17]. In this follow-up study, we endeavored to further explore the potential beneficial effect of EXQ on two constipated mice models, named HHPD model and HHPD + atropine model, which mimicked pediatric constipation due to HHPD and in combination with poor gastrointestinal mobility. Results revealed that the HHPD and HHPD + atropine groups showed evident constipation symptoms, including significantly decreased fecal number, fecal weight and increased intestinal contents due to inhibited colonic peristalsis in comparison to control group. Whereas, EXQ treatment dose-dependently relieved these symptoms. The prokinetic and laxative effects of EXQ against HHPD-induced and HHPD + atropine induced constipation might be closely related to the promotion of intestinal motility.

As reported, immunity and inflammation were intimately associated with chronic constipation [38]. Thymus and spleen are two major immune organs that crucially involved in the immunological responses. The spleen and thymus indexes usually depend on the lymphocyte proliferation [39]. Hence, in the present work, we examined the two indexes to roughly evaluate the immune function [30, 39]. In the HHPD model test, the vehicle treatment significantly elevated the thymus and spleen indexes as compared with the normal control, indicating an activated immune response in HHPD-treated mice. This observation was consistent with previous report which revealed the correlation between low-grade intestinal inflammation and constipation [18]. EXQ treatment significantly decreased the thymus and spleen indexes when compared to the HHPD vehicle, indicating that EXQ mitigated the immunological and inflammatory response in constipation mice model.

However, in HHPD + atropine model test, the thymus index of constipated mice decreased, although the spleen index increased significantly. The inconsistent trend of variations in thymus and spleen indexes was also observed in other animal models concerning immunity [40–42]. Whereas, EXQ treatment observably reversed the changes in HHPD + atropine mice by decreasing the elevated spleen index and raising the depressed thymus index, suggesting that EXQ might modulate the immune function to a more favorable balance. However, more investigations are merited to gain further insight into the detailed mechanism.

Additionally, sIgA is the main local immunoglobulin found in gastrointestinal secretions [31, 32, 43], which protects mucosal surfaces from microbial invaders and inhibits their attachment to the epithelium [31]. In this study, the colonic level of sIgA was decreased significantly in HHPD vehicle group, which led to weakened colonic immunity. Conversely, EXQ treatment markedly increased the sIgA level in a dose-dependent manner, suggesting the enhanced immunity function on the intestinal barrier, which might resist potential damage triggered by HHPD. A similar trend of change was observed in HHPD + atropine model test, however, the data failed to reach significant difference, perhaps due to limited sample size (*n* = 8 in each group). The result was consistent with the microscopic observation after H&E staining, which also supported that EXQ effectively increased the integrity of colonic mucosa as compared to the vehicle group [29].

Chronic constipation is characteristic of low-grade inflammation in mucosa [18]. Correspondingly, colonic inflammation frequently causes dysmotility and impairs propulsion [20, 21]. Thus, constipation and concurrent colonic damage usually reciprocally exacerbates each other in pathological conditions. The consumption of HHPD could induce the proliferation of harmful bacteria and imbalance in the intestinal microflora, then affecting the intestinal epithelial barrier [44, 45]. Under this condition, endotoxins like LPS might permeate to the systemic compartment as a consequence of a disturbed gastrointestinal barrier function, stimulate the macrophages to produce pro-inflammatory cytokines, and triggered systemic inflammation [46, 47]. There are pro-inflammatory cytokines such as TNF-α, which promoted tissue repairment, mainly mediated inflammatory reaction and caused tissue damage at a high level; and anti-inflammatory cytokines such as IL-10 [48, 49], mainly inhibited the release of inflammatory mediators [50]. In current study, elevated productions of colonic TNF-α and IL-10 were observed in HHPD-induced constipated mice. And EXQ treatment markedly decreased the levels of IL-10 and TNF-α, suggesting the ameliorative effect of EXQ on constipated mice was associated with its anti-inflammatory activity against mucosa inflammation. In HHPD + atropine model test, the levels of colonic and serum LPS were remarkably increased, and the intestinal epithelial cells were irregularly arranged, accompanied with inflammatory response. These results strongly indicated that constipation could damage the intestinal epithelial barrier, and thus increased detrimental bacterium might invade the colonic tissue and come into circulation. Whereas, EXQ significantly decreased the colonic and serum LPS, and improved the colonic morphology. The attenuation on inflammatory response might be associated with lower intestinal permeability and improvement on the intestinal cellular barrier [47]. Therefore, the above data suggested that EXQ effectively improved the deteriorating histological changes in constipated mice, and protected mice against mucosal injuries induced by HHPD + atropine [14].

As the main constituent of EXQ, *Semen Coicis* was proved to exhibit promising protective effects on gastrointestinal tract by regulating gut microbiota and attenuating low-grade inflammation [33, 51]. *Fructus crataegi* was reported to relieve dyspepsia and beneficially promote gastrointestinal motility [52]. The water extract of *Fructus crataegi* exhibited remarkable antagonism to atropine challenge, suggesting that *Fructus crataegi* probably worked on gastrointestinal M-receptor, released acetyl choline and improved the gastrointestinal peristalsis [53, 54]. Besides, *Fructus crataegi* was shown to improve the cell-mediated immunity and humoral immunity by increasing the spleen and thymus weight, T lymphocyte cell transformation rate and so on [53]. Therefore, the immune-regulating and anti-constipation effect may attribute to these components.

Further investigations are guaranteed to reveal the active fractions and gain in-depth insights into the underlying mechanisms.

## Conclusion

In summary, this study revealed that EXQ effectively alleviated constipation by improving fecal output, alleviating the concomitant inflammatory responses by enhancing sIgA level, and reducing the levels of IL-10, TNF-α and LPS in constipated mice induced by HHPD and HHPD + atropine. The overall protective mechanism of EXQ against HHPD and insufficient gastrointestinal mobility suggested a synthetic effect involving laxation, modulation of inflammatory statuses and protection on intestinal barrier. Our results corroborated its conventional indications and validated its laxative activity, lending more credence to the clinical application of EXQ in pediatric constipation related symptoms.

## Abbreviations

ELISA: Enzyme Linked Immunosorbent Assay
EXQ: Xiao’er Qixingcha
HHPD: High-heat and High-protein diet
HPLC: High Performance Liquid Chromatography
IL-10: Interleukin-10
LPS: Lipopolysaccharide
PBS: Phosphate Buffered Saline
sIgA: Secretory Immunoglobulin A
TNF-α: Necrosis Factor-α
